# Facile Quantification and Identification Techniques for Reducing Gases over a Wide Concentration Range Using a MOS Sensor in Temperature-Cycled Operation

**DOI:** 10.3390/s18030744

**Published:** 2018-03-01

**Authors:** Caroline Schultealbert, Tobias Baur, Andreas Schütze, Tilman Sauerwald

**Affiliations:** Lab for Measurement Technology, Saarland University, 66123 Saarbrücken, Germany; t.baur@lmt.uni-saarland.de (T.B.); schuetze@lmt.uni-saarland.de (A.S.); t.sauerwald@lmt.uni-saarland.de (T.S.)

**Keywords:** MOS gas sensor, temperature-cycled operation, differential surface reduction, quantification, sensitivity, selectivity, linear calibration

## Abstract

Dedicated methods for quantification and identification of reducing gases based on model-based temperature-cycled operation (TCO) using a single commercial MOS gas sensor are presented. During high temperature phases the sensor surface is highly oxidized, yielding a significant sensitivity increase after switching to lower temperatures (differential surface reduction, DSR). For low concentrations, the slope of the logarithmic conductance during this low-temperature phase is evaluated and can directly be used for quantification. For higher concentrations, the time constant for reaching a stable conductance during the same low-temperature phase is evaluated. Both signals represent the reaction rate of the reducing gas on the strongly oxidized surface at this low temperature and provide a linear calibration curve, which is exceptional for MOS sensors. By determining these reaction rates on different low-temperature plateaus and applying pattern recognition, the resulting footprint can be used for identification of different gases. All methods are tested over a wide concentration range from 10 ppb to 100 ppm (4 orders of magnitude) for four different reducing gases (CO, H_2_, ammonia and benzene) using randomized gas exposures.

## 1. Introduction

Metal oxide semiconductor (MOS) gas sensors are widely used for the detection of reducing and oxidizing gases. Applications are reported in many fields, e.g., the detection of explosive gases [1,2], fire detection [3,4], odour monitoring [5] and air quality control [6,7]. Accordingly, a sensor should be capable of measuring a large variety of gases over a broad range of concentrations.

For the identification of different gases either a multisensor array [8] or a virtual multisensor, e.g., using temperature-cycled operation (TCO) [3,9,10], is set up and pattern recognition methods are applied [11]. TCO dates back more than 40 years, utilized for the discrimination of carbon monoxide and hydrocarbons [12]. It used, as most of the early attempts, a heuristically defined temperature cycle and only very few virtual multi-sensors, e.g., on temperature plateaus, instead of using an optimized cycle and virtual multi-sensor generation. This is probably due to the fact that modelling the sensor response throughout a cycle is quite challenging. The relaxation of surface-state occupancy can be relatively slow (cf. [13]) compared to practical temperature cycle and, therefore, a model of the sensor response needs to address the transient effects of one or more surface species throughout the full temperature range. While the observation of the temperature-dependant rate constants is limited for the classical Taguchi-type sensors due to their high thermal time constants, micro-machined sensor allowed a more comprehensive study of the transient sensor response, including determination of rate constants for several extrinsic and intrinsic surface states [14]. Despite the limited ability to determine the rate constant for Taguchi-type sensors, a rate constant-based model had been used to describe a method for the generation of virtual sensor from TCO data [15]. Following the line of the rate equation approach by Ding et al. [14], we described a concept for the optimization of TCO and feature generation in earlier work [16,17], which gives the basis for the quantification technique presented in this paper.

Especially for isothermal sensors [18,19] but as well for some sensor with TCO [20] calibration curves for quantification are in the form of a power law even if multivariate methods are sometimes needed [21]. A facile and preferably linear method for the quantification of gases independent from environmental impacts like humidity can contribute to practical applications of MOS gas sensors. The method should be free of complex algorithms to keep computational effort as low as possible.

In the past, we developed a model for the conductance of a SnO_2_-based MOS sensor during specific transient states of a temperature cycle [17], where the sensor shows improved sensitivity [22]. In this paper, we demonstrate two facile methods allowing linear calibration of gas sensors that are derived from this model and demonstrate their suitability by measuring four gases (CO, H_2_, ammonia and benzene) over four decades of concentration (10 ppb to 100 ppm). The identification of these gases using supervised pattern recognition (LDA, linear discriminant analysis) based on the parameters extracted for quantification and calculated on different temperature levels is also shown.

The mentioned model is based on the conductance of grain–grain boundaries, which is the dominating effect for a SnO_2_ layer. The conductance of these boundaries (Equation (1)) is dominated by a negatively charged surface state, causing an energy barrier $E_{B}$ between grains where the height of the barrier is a quadratic function of the density of these surface states $N_{S}$:

(1) $$G=G_{0}\cdot e^{-\frac{E_{B}}{k_{B}T}}\;\wedge \;E_{B}\propto N_{S}{}^{2}$$

Please note that Equation (1) does not specify the surface state and indeed several types of intrinsic [14] and extrinsic [23], mainly oxygen-related surface states, have been reported and discussed in literature. The interplay of the extrinsic oxygen-related surface states is still discussed in literature [24,25,26] especially with respect to changing sensor temperature and surrounding conditions. At lower temperatures, $O_{2}^{-}$ is often found to be the dominating species being replaced by $O^{-}$ for higher temperatures (e.g., above 700 K in [26]). Some models also include $O^{2-}$ in this higher-temperature region [27] and an interplay with humidity, as in humid air the adsorption of $O^{2-}$ is reported to be blocked and $O^{-}$ becomes dominant [28]. Despite the high variety in possible surface states, we could show in earlier work that for the specific operation mode used in this paper it is found to be sufficient to assume one single dominating ionosorbed species on the surface and single-step reaction with reducing gas [17]. This operation mode will shortly be described in the following section.

The reaction of a reducing gas with this reactive species reduces the energy barrier and increases the conductance strongly. We have reported that the energy barrier, and thereby the surface coverage of the semiconductor with negative surface charge, are temperature-dependent [22]. In the temperature range from 130 °C to 420 °C a strong increase can be observed, which in turn results in a gas-specific temperature profile allowing identification of different gases by temperature-cycled operation [9,10]. However, temperature cycling must not be understood as a sequence of equilibrium measurements of the sensor conductance at different temperature as the time constant for equilibration of ionosorbed surface species is typically higher than the duration of the temperature cycle. Ding et al. have demonstrated that a set of rate equations for adsorption and desorption of surface states can be used to describe the profile of the conductance throughout the temperature cycle [14]. Following this observation, we have shown that an excess of negative surface charge coverage can be obtained with a fast temperature decrease, which can be utilized for a strong enhancement of the sensor response and sensitivity [22]. After the fast temperature reduction, the sensor surface is far from equilibrium with a large excess of negative surface charge and the rate constant for equilibration is very small due to the low temperature [17]. The sensor surface is then predominantly reduced by desorption of excess surface states. We have shown that a simple model with only one rate equation for this desorption is sufficient to describe the surface coverage and, with Equation (1), the conductance shortly after the temperature reduction is described by

(2) $$\frac{dN_{S}}{dt}=-k\left(c_{gas}\right)N_{S}$$

In this equation the rate constant *k* is a linear function of the gas concentration $c_{gas}$. Using Equation (1) the change of the logarithmic sensor conductance at the beginning of a low temperature becomes

(3) $$\frac{\mathrm{dln}\left(G\left(t\right)\right)}{dt}\sim k\left(c\right)=k_{gas}c_{gas}+k_{0}$$

The constant term $k_{0}$ subsumes the desorption of negative surface charge without gas reaction and the desorption caused by traces of reducing gases in the clean air background. Please note that Equation (2) is only valid if the surface charge is still well above equilibrium, otherwise the inverse reaction, that is, adsorption of new surface charge, is not negligible anymore. While we could demonstrate that for small gas concentrations a calculation of the rate constant by a linear fit of ln(G) shortly after the temperature reduction is a suitable method for estimating the rate constant, it is obvious that this method fails when applied over a wide range of concentrations. In the following we will present a facile method for estimation of the rate constant and therefore quantification of gases over a wide range of concentrations.

## 2. Materials and Methods

Data treatment is kept as simple as possible and was performed with the toolbox DAV^3^E, which is a software developed at our institute [29]. To cover the high dynamic concentration range, two methods (i–ii) for quantification are used:

(i). For the quantification of low concentrations when the sensor does not approach equilibrium of surface coverage during the low-temperature phase, the rate constant is estimated by the calculation of $\tilde{k}$ which denotes the slope of ln(G(t)). Following the line of [17], $\tilde{k}$ is calculated by a linear fit of ln(G(t)) at the beginning of the low-temperature range. According to Equation (3), $\tilde{k}$ is proportional to the reaction rate constant k and, furthermore, linear to the concentration c [17]. The proportionality factor f between k and $\tilde{k}$ can be calculated using the initial surface coverage at low temperature [17]:
  (4) $$f=E_{B}\left(0\right)\cdot 2k_{B}T_{high}\;\wedge \;E_{B}\left(0\right)=k_{B}\ln\left(\frac{G_{high}}{G_{low}}\right)\cdot \frac{T_{high}T_{low}}{T_{high}-T_{low}}$$
  However, as the calculated feature $\tilde{k}$ is calibrated with the measurement results and the calculation is much more complex than one single linear fit, the explicit calculation of k will be omitted for most of the measurements except for one explicit comparison in Section 4. To compensate the background, the slope of zero air ${\tilde{k}}_{0}=fk_{0}$ is subtracted from all calculated values [17].
(ii). For the quantification of high concentrations when the sensor almost reaches equilibrium surface coverage during the low-temperature phase, the time constant of the relaxation process is evaluated by taking the highest and lowest values of ln(G) and evaluating the time-constant τ for reaching 63.2%=1−1/e of the difference using the MATLAB function *interp1*:
  (5) $$\tau =t\left(0.632\cdot \left(\ln\left(G_{max}\right)-\ln\left(G_{min}\right)\right)\right)-t_{start}$$
  According to the solution of the simple differential Equation (3), the inverse value 1/τ equals 2k and is therefore linear to c. For practical reasons, the constant $k_{0}$ can be omitted as it is negligible in the high concentration range compared to $k_{gas}c_{gas}$. Therefore, the signal 1/τ will be proportional to the gas concentration.

For the investigations a commercially available SnO_2_-based MOS gas sensor (AS-MLV-P2, ams Sensor Solutions Germany GmbH) is used. During the TCO, the sensor is periodically oxidized at a high temperature so that the reduction of the oxygen excess at a lower temperature can be analyzed as proposed. The full cycle consists of three of these temperature steps, each starting with 3 s at 450 °C followed by 27 s at a lower temperature (150 °C, 200 °C and 250 °C), resulting in a total duration of 90 s.

The concentration at which the method needs to be switched depends on the sensitivity of the sensor to the given gas at the considered temperature. We decided to switch the methods when the sensor reaches a stable value during the 27 s long low-temperature phase (concentration values for the method switching can be found in Table 1), which can also be implemented as an automatic evaluation algorithm. For the quantification, one single temperature step is sufficient, we chose the lowest one (150 °C) which, according to the presented model, should provide the highest sensitivity. For the identification of reducing gases, all three temperature steps are evaluated (cf. Section 3.3). The linear fit for the calculation of $\tilde{k}$ according to (i) is always performed between 10 and 20 s. For the τ-evaluation (ii), the whole plateau from 3 to 30 s is considered. The identification using several temperature steps is demonstrated by $\tilde{k}$-evaluation, the additional ranges for the calculation of $\tilde{k}$ are 34–36 s and 64–65 s, they are much closer to the temperature change because of the much faster relaxation at the higher temperatures (200 °C and 250 °C, respectively).

The electronic system basically consists of two parts: temperature control and sensitive layer read-out (specific information can be found in [30]). The heater was calibrated according to a power-to-temperature curve provided by the manufacturer. We transform this power–temperature curve to a resistance–temperature curve by a calibration measurement. The heater is then controlled via a closed-loop control. A stable temperature is reached after approximately 140 ms (see Supplementary Material Figure S1) The read-out is based on a logarithmic amplifier, which is placed directly at the sensor to allow low noise amplification of the low currents induced by a constant voltage over the sensing layer. Data acquisition is performed by a Teensy, a microcontroller system similar to Arduino, with a sample rate of 1 kHz. Before applying any data treatment, the signal quality is enhanced by averaging with n=20. No further data treatment is needed, as both presented methods are based on the logarithmic conductance ln(G), which means that the recorded ADC-values can be directly used for all evaluations. However, the value of *G* can be calculated according to Equation (6), the measured values during the measurements are in the range of 300 kΩ and 6 GΩ

(6) $$G=249\cdot 10^{2}*10^{\frac{-6.3574\cdot ADC}{2^{16}}+4.65367}$$

The gas profile is generated by our gas mixing system (GMA) operating with dynamic dilution. The system consisting of several mass flow controllers (MFCs) and valves has been described in detail in [31]. The carrier gas is mixed by two MFCs with a maximum flow of 500 mL/min each, one with dry zero air (provided by a ULTRA zero air generator with catalytic conversion and a dew point of −70 °C) and one humidified by leading the zero air through an isothermal water bubbler at 20 °C. The relative humidity (RH) can be set through the mixing ratio of these two flows and the flows of the dry test gases. The temperature of the laboratory, thus the temperature in the sensor chamber, is approximately 22.5 °C, which means that the relative humidity at the sensor is slightly lower than set. The system provides four pre-dilution lines for gases from pressure cylinders, which means that the gas from the cylinder is diluted in two steps: first via two MFCs (500 mL/min and 10 or 20 mL/min respectively) and then in a second step by adding it to the carrier gas flow via a valve with a 10 or 20 mL/min MFC. There are two lines with 10 mL/min and two with 20 mL/min MFCs, giving possible dilution factors of 500,000 and 125,000, respectively, for this investigation, which used a total carrier gas flow of 250 mL/min for all experiments. Using three-way valves, the dilution line can be set to the desired concentration to reach equilibrium before the gas mixture is actually offered to the sensor. The sensor is connected to the GMA via a stainless-steel block with one drill hole for the flow (diameter ca. 8 mm) and an orthogonal drill hole with the diameter of the TO cap of the sensor. The distance between the top of the sensor housing and the gas flow is approximately 4 mm.

Four gases (carbon monoxide CO, $c_{cylinder}=1995\;\mathrm{ppm}$, 10 mL/min dilution line; hydrogen H_2_, $c_{cylinder}=104\;\mathrm{ppm}$, 20 mL/min dilution line; ammonia NH_3_, $c_{cylinder}=2903\;\mathrm{ppm}$, 10 mL/min dilution line; benzene C_6_H_6_, $c_{cylinder}=99.2\;\mathrm{ppm}$, 20 mL/min dilution line) are used to verify the model. Three concentrations per decade (10, 25, 50, 100, 250, 500 ppb, etc.) have been offered starting at 10 ppb and ending at the highest possible concentration (see Table 1). Each concentration is offered for 30 min to the sensor followed by pure zero air for 10 min. The dilution line configuration for the next planned gas and concentration is already set at the beginning of the preceding one, which means 40 min for reaching equilibrium inside each dilution line. Gases are always offered in the same order: NH_3_, H_2_, CO, C_6_H_6_. The concentration order is randomized using the MATLAB function *randperm* to prevent time-dependent effects from affecting the quantification method. The full profile is performed at two humidities (40% RH and 50% RH) to investigate the influence of humidities.

## 3. Results

### 3.1. Overview

**Figure 1:**
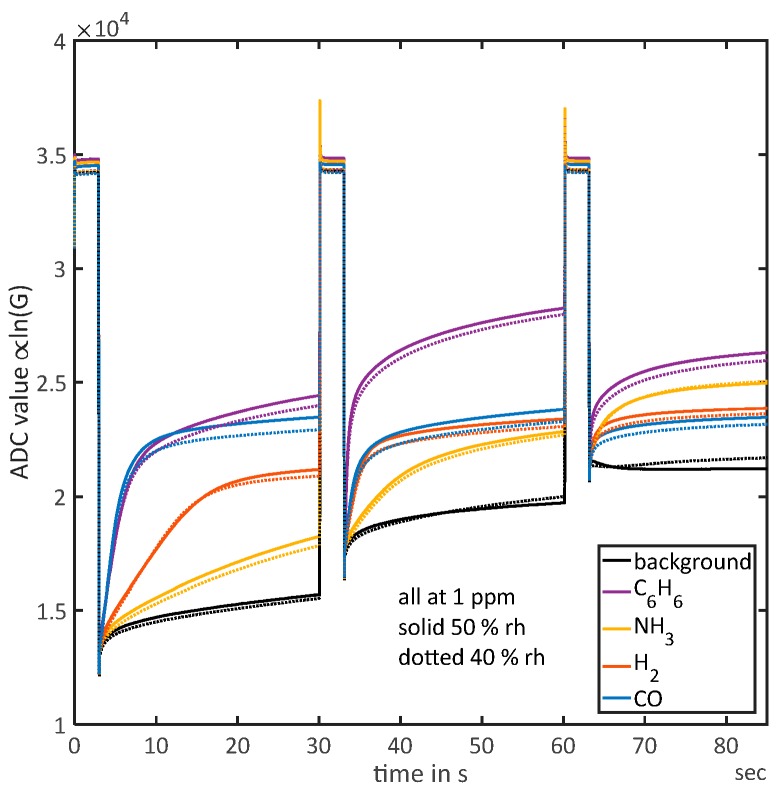
Sensor signal for all gases at 1 ppm and background gas (zero air) at both humidities measured (40% RH and 50% RH), the measured ADC-values are proportional to the logarithmic conductance due to the logarithmic amplifier. Temperatures: 0–3 s 450 °C, 3–30 s 150 °C, 30–33 s 450 °C, 33–60 s 200 °C, 60–63 s 450 °C and 63–90 s 250 °C.

In Figure 1, full cycles for 1 ppm of each gas are shown at different humidities. It can be observed that humidity influences the absolute value of ln(G) but the shape of the ln(G) curves are almost parallel for the two tested humidities. Only on the 250 °C plateau a significant deviation of the shape can be observed for background air. The suggested quantification methods evaluating the shape of the signal should, therefore, not be affected by humidity. Figure 1 also shows that different gases show different sensitivities: while the ammonia signal (yellow) has not reached equilibrium during the first low-temperature phase and the slope-method can be applied, the other gases all show a linear response initially but quickly run in during this phase. While H_2_ and CO show nearly constant values after the first linear response, that is, have reached equilibrium, Benzene shows a second relaxation process with a higher time constant, so no constant value is reached during that temperature plateau.

**Figure 2:**
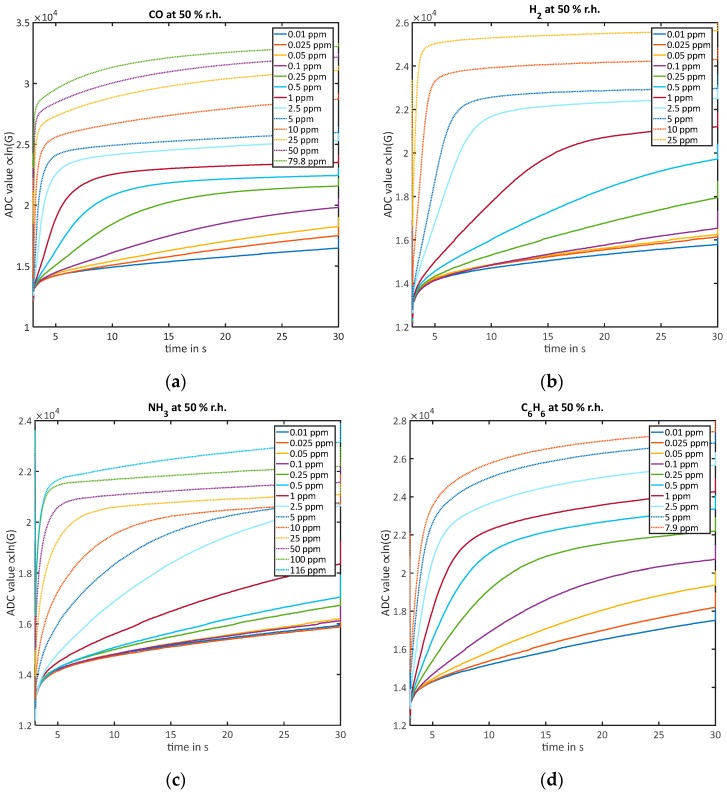
Measured ADC signal which is proportional to the logarithmic conductance ln(G) during the 150 °C temperature phase (3–30 s during the cycle) at 50% RH for all measured concentrations: (**a**) CO; (**b**) H_2_; (**c**) NH_3_; (**d**) C_6_H_6_.

Figure 2 gives an overview of the low-temperature phase at 150 °C starting at 3 s relevant for the quantification at 50% RH for all gases and all measured concentrations. For the data analysis, we always used the last cycle of each gas exposure to ensure that the concentration has reached a stable value inside the sensor chamber. For low concentrations, all gases can be quantified using the $\tilde{k}$-evaluation. Curves ascending with the concentration can be clearly observed for CO, H_2_ and benzene. For ammonia, the absolute values at low concentrations show a wrong order: the curve for 10 ppb is above that for 25 ppb and 50 ppb is above 100 ppb. This wrong order is also observed for the $\tilde{k}$-evaluation. The GMA is completely set up with stainless steel and ammonia is known to adsorb on such surfaces [32], which could explain this inconsistency, because the two displaced concentrations 10 ppb and 50 ppb followed after 50 ppm and 116 ppm ammonia. Looking at the higher concentrations, the faster relaxation and reaching of equilibrium can be observed very well for all gases. Similar to the benzene cycle in Figure 1, the highest concentrations of CO and ammonia as well as H_2_ (although the effect is very small here) show a second time constant with a further slight increase of ln(G) after the linear range has ended and equilibrium should be reached.

### 3.2. Quantification

#### 3.2.1. Quantification of Low Concentrations without Reaching Equilibrium during the Low-Temperature Phase ($\tilde{k}$)

**Figure 3:**
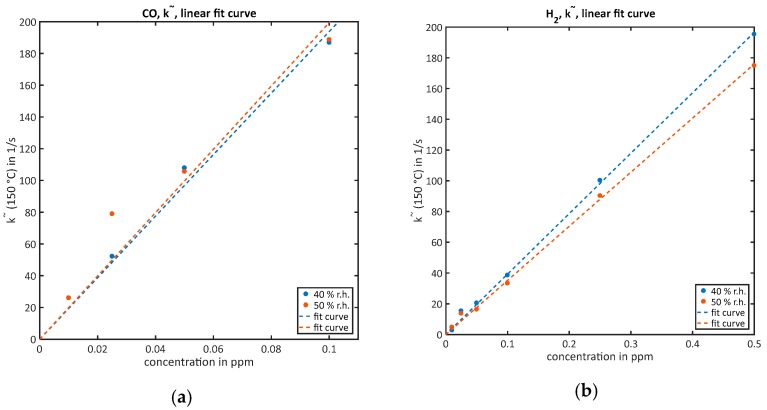

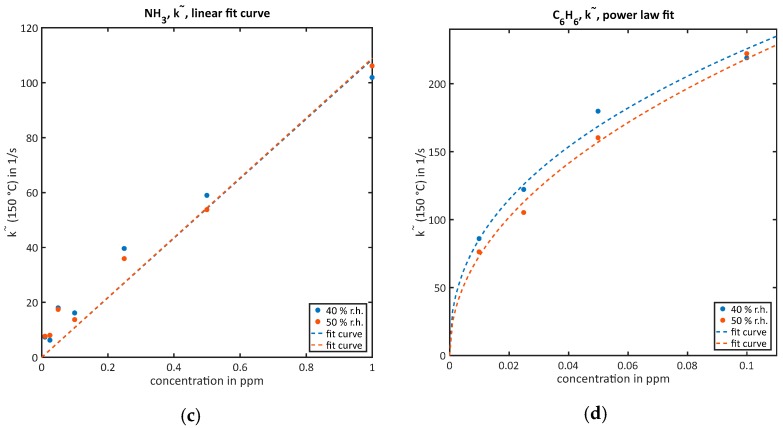
Quantification of the correspondent gases via the $\tilde{k}$-evaluation and their fit curves for both humidities: (**a**) CO, linear fit curve; (**b**) H_2_, linear fit curve; (**c**) NH_3_, linear fit curve; (**d**) C_6_H_6_, power law fit.

For low concentrations, the $\tilde{k}$-evaluation was tested for quantification. The goal of this treatment is to achieve a linear calibration curve. Since the zero air ${\tilde{k}}_{0}$ has been subtracted from all computed $\tilde{k}$, the expected function is a line through the origin, so fitting was performed using the very simple function f(x)=a⋅x. The results for all gases and both humidities can be seen in Figure 3; all obtained fit parameters are summarized in Table 2. Figure 3a shows the results for CO: for 40% RH, $a_{40}$ is 1939; for 50% RH, $a_{50}=1995$. Both values lie within the 95% confidence bounds of each other. The coefficients of determination $R_{40}^{2}$ are 0.9851 and 0.9252, respectively. The low value of $R_{50}^{2}$ is mainly caused by the point at 25 ppb, which was the very first CO concentration in this measurement. The results for hydrogen, shown in Figure 3b, are $a_{40}=393.1$ and $a_{50}=351.9$, $R_{40}^{2}=0.9986$ and $R_{50}^{2}=0.9983$. In this case, the values for a are not within the confidence interval of each other, but the quality of both fits is very high. For ammonia (Figure 3c), fitting quality is lower than for the other gases, which could be already expected from the raw data in Figure 2c. We get $a_{40}=108.5$, $a_{50}=108.9$, $R_{40}^{2}=0.9381$ and $R_{50}^{2}=0.9606$. Only for benzene (Figure 3d) a linear fit was not possible. In this case a power law $f\left(x\right)=a\cdot x^{b}$ represents the data much better, resulting in $a_{40}=569.9$ and $b_{40}=0.4065$, $a_{50}=691.1$ and $b_{50}=0.4935$. The fit quality is $R_{40}^{2}=0.9835$ and $R_{50}^{2}=0.9938$.

#### 3.2.2. Quantification of High Concentrations While Reaching Equilibrium during the Low-Temperature Phase (τ)

**Figure 4:**
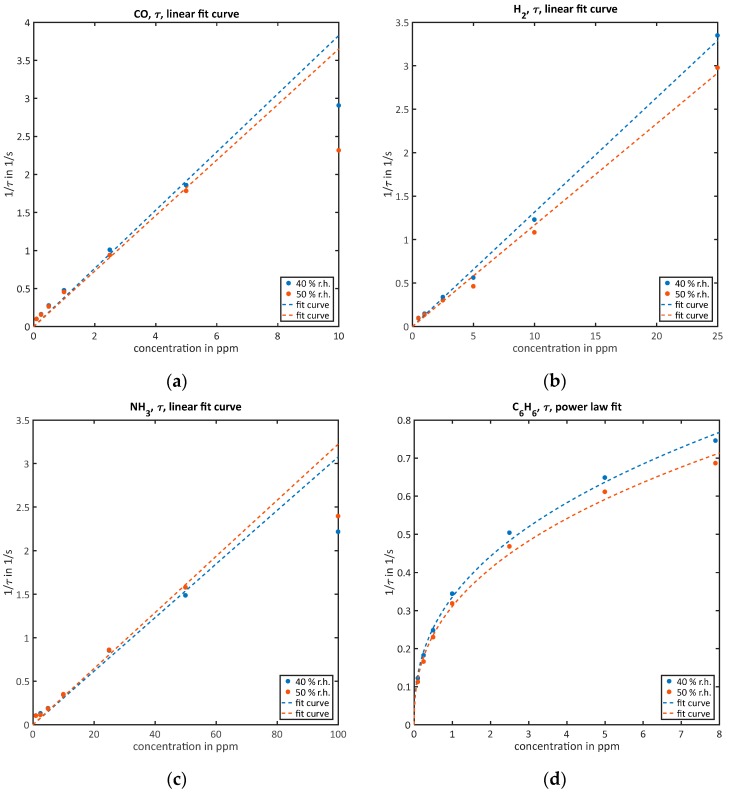
Quantification of the correspondent gases via the τ-evaluation and their fit curves for both humidities: (**a**) CO, linear fit curve; (**b**) H_2_, linear fit curve; (**c**) NH_3_, linear fit curve; (**d**) C_6_H_6_, power law fit.

For higher concentrations starting with the highest one used in the last Section (3.2.1), the τ-evaluation is used for quantification. Again, the calculated values 1/τ over corresponding concentrations are shown in Figure 4 for all gases together with the corresponding fit curves, which are again straight lines through the origin for CO, H_2_ and ammonia and a power law for benzene. The fit parameters can be found in Table 3. For CO, in Figure 4a the obtained results are: $a_{40}=0.3827$ and $a_{50}=0.365$, both results are again inside the respective confidence intervals, and $R_{40}^{2}=0.9874$ and $R_{50}^{2}=0.9883$. For H_2,_ in Figure 4b the obtained results are: $a_{40}=0.1317$ and $a_{50}=0.1167$, as before for H_2_ the results for different humidities are outside the confidence intervals, but the fit quality again is very high with $R_{40}^{2}=0.9972$ and $R_{50}^{2}=0.9955$. The higher ammonia concentrations evaluated here follow the expected model much better than the lower ones shown above. We get (Figure 4c) $a_{40}=0.03078$ and $a_{50}=0.03224$, both results are inside the confidence intervals, and the corresponding coefficients of determination $R_{40}^{2}=0.9888$ and $R_{50}^{2}=0.996$. Also, for this method and the higher benzene concentrations it is not possible to perform a linear approximation as shown in Figure 4d. Again, a power law is used instead, yielding: $a_{40}=0.3358$ and $a_{50}=0.3114$, $b_{40}=0.3977$ and $b_{50}=0.3987$, almost all values are inside the confidence intervals of each other, except $a_{40}$. The fit quality again is high, $R_{40}^{2}=0.9963$ and $R_{50}^{2}=0.9942$.

The highest concentrations of CO (10 ppm) and ammonia (100 ppm) in Figure 4 show significant deviations from the fit curves and were not used for the approximation. This is caused by the strong effect of the observed second time constant after the relaxation considered in the model is finished. The very simple quantification rule used here is not able to compensate this effect. Therefore, higher concentrations above 10 ppm CO and 100 ppm ammonia cannot be evaluated with the presented method.

### 3.3. Identification

Looking back to Figure 1, it becomes clear that the four gases can be distinguished based on their different reaction rates at the three temperatures. At 150 °C, CO and benzene show similar reactions rates, but for both higher temperatures benzene exceeds CO clearly with CO even showing the lowest rate at 250 °C. Ammonia always shows the lowest reaction rate except at the highest temperature of 300 °C, where it shows the second strongest response after benzene. For all temperatures the reaction to H_2_ is between the other gases.

**Figure 5:**
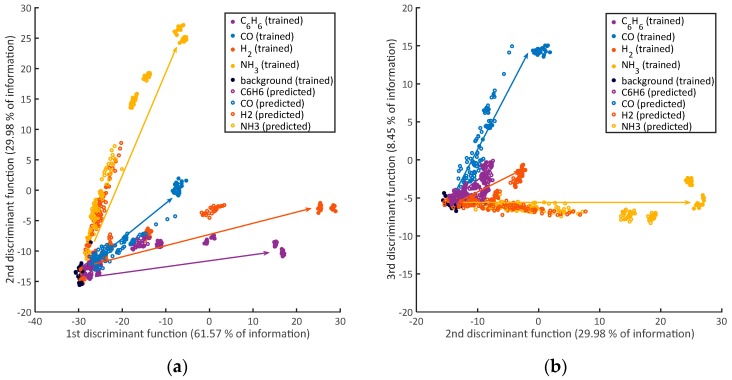
A 3D-LDA (Linear Discriminant Analysis) scatterplot trained (filled circles) by background air and the highest concentration from the $\tilde{k}$-evaluation of each gas, all other lower concentrations are predicted (empty circles), each gas has its own direction to step out of the background air data. The $\tilde{k}$ for the three different low temperatures have been used as features; (**a**) shows the first two discriminant functions; (**b**) the second and third.

Pattern recognition is needed for this type of analysis. We chose to show the possibility of identification with model-based TCO and model-based data evaluation with Linear Discriminant Analysis (LDA). This method projects the data into a new often lower dimensional feature space while maintaining as much relevant information as possible [33]. For this, the coefficients for the linear feature combinations are chosen in order to minimize the within-class scatter while maximising the between-class scatter in the new discriminant space. As features we use the three $\tilde{k}$ calculated for the three low temperatures. For training we use all measured temperature cycles at the highest concentrations of each gas (12 per humidity), which can be correctly quantified using the $\tilde{k}$-evaluation (CO 0.1 ppm, H_2_ 1 ppm, NH_3_ 1 ppm, C_6_H_6_ 0.1 ppm) and background gas, all at both humidities. To compensate for unequal class sizes, selections are removed randomly from larger classes before LDA, resulting in a total of 120 observations (temperature cycles) for the training dataset (24 per class). We use three discriminant functions and a 10-fold cross-validation [33], the error from this validation for the training dataset is 0%. After the training, all lower concentrations are predicted using the obtained model, as shown in Figure 5a for the first two discriminant functions and Figure 5b for the second and third discriminant functions. Filled circles are trained observations, empty ones predicted observations. It can be seen that all lower concentrations start near the trained background observations (black) and then scatter in the direction (arrows) of the corresponding highest concentration which has been trained. Especially ammonia and benzene can be distinguished clearly in Figure 5a. For CO and H_2_, the third discriminant function needs to be considered, which still contains 8.45% of the relevant information. In Figure 5a, few of the red empty circles (H_2_) scatter in the direction of ammonia. Tracing back those points, it becomes clear that these are low H_2_ concentrations which directly follow high ammonia concentrations, for example, the confusion is presumably caused by carry-over in the GMA due to adsorption of ammonia on the internal surfaces.

## 4. Discussion

**Figure 6:**
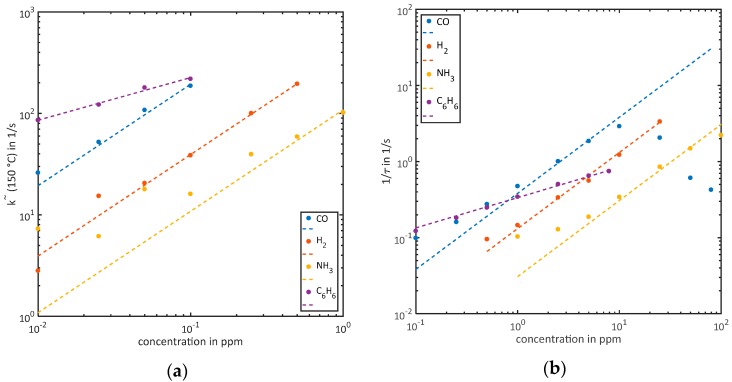
The results from Section 3.2 on a double logarithmic scale and for all gases in one graph: (**a**) $\tilde{k}$-evaluation for low concentrations showing the very high response of benzene compared to the other gases; (**b**) τ-evaluation for the higher concentrations, here the response of benzene is below the others because of the different correlation between concentration and sensor signal.

A comparison of all four investigated gases is given in Figure 6a for the $\tilde{k}$-evaluation and Figure 6b for the τ-evaluation. Both graphs are on a double logarithmic scale to give a better overview. A linear calibration curve is exceptional for MOS gas sensors, although we see that the relative error for both evaluations increases in the lower concentration range. This can be avoided by applying a logarithm to x- and y-data before performing the fit (function: f(x)=log(a)+x), because then points from every order of magnitude have the same influence on the fitting quality. With this approach, of course the absolute error for the higher concentrations increases. The poorest agreement between fit and data points is observed for low ammonia concentrations in Figure 6a. Applying a linear fit with an offset (function: f(x)=a⋅x+b) improves the fit notably. The reason for this could be a systematic error in the generation of small ammonia concentrations, giving an additional background compared to the zero air. Ammonia is known to adsorb easily on stainless steel surfaces, an additional error can therefore be expected, caused by ad- and desorption of ammonia inside the dilution line [32].

Using the presented quantification methods on several temperatures, an identification of the gas can be achieved rather easily directly upon the observed differences in reaction kinetics.

In Figure 6, benzene immediately sticks out because of its very different behavior. Despite the expectations from the model and in contrast to the other gases, the benzene data is not represented by a linear calibration curve but by a power law with an exponent of approx. 0.4. In previous works, we also found similar results for gases with aromatic structures, especially toluene [17]. The simple model assumes that there is no competition for adsorption sites on the surface (Henry adsorption), no interaction between molecules and only one surface species for the reaction. For aromatic substances, it is likely that a precursor is needed to crack the ring structure resulting in a Freundlich isotherm, which would suit the power law. The chemical products resulting from the precursor reaction tend to have very low vapor pressures and thus stay on the surface for a long time, as we have already observed when measuring short trace gas pulses [16], which explains the very high sensitivity at low concentrations compared to the other gases, because of possible multi-stage reaction processes.

For the other gases, a linear quantification using the two simple methods presented here is clearly possible. There are distinct concentration ranges where each method is valid with the boundaries depending on the sensitivity of the respective gas at the given temperature. From Figure 6a, the upper limit for the slope-evaluation can be estimated at slope value of about 110–120 1/s. Similarly, the validity of the τ-method ends at approx. 1.2 1/s for all gases; at higher values the effect of the second time constant observed in Figure 2 limits the performance of the simple algorithm. A more complex algorithm for the linear approximation and calculation of τ would overcome this limitation. We also tested evaluating the time required to reach a certain threshold level starting from $\ln\left(G_{min}\right)$, which all concentrations reach inside the 27 s; this is also suitable for quantification over the full range of concentrations but does not yield a linear calibration curve. The slow increase in conductance with a second time constant observed mainly in the ammonia and CO measurements is not covered by the presented model. For the description of the effect, a multitude of possible additions to the model could be discussed, ranging from the impact of adsorbed reducing species [34] to a gradual reduction of the sensing materials due to a change in oxygen vacancies (bulk donors) equilibrium density $N_{D}$ [35]. However, the effect is small compared to the total relaxation and the two time constants differ strongly so they can clearly be discriminated by the naked eye. Therefore, an optimization of the feature extraction method is likely to be the best solution.

To apply one single calibration curve, the values for $\tilde{k}$ and 1/τ must be converted to k, which is possible using Equation (4) and 2k=1/τ. We tested this conversion for H_2_ at 2.5 ppm, where the linear fit needs to be reduced to the first 3 s after the temperature change and τ-evaluation works properly. We obtained $k\left({\tilde{k}}_{40}\right)=0.0282\frac{1}{s}$, $k\left({\tilde{k}}_{50}\right)=0.0256\frac{1}{s}$ and $k\left(\tau_{40}\right)=0.1682\frac{1}{s}$, $k\left(\tau_{50}\right)=0.1501\frac{1}{s}$; thus, a factor of approximately 6 between both methods. The model used here is probably not considering additional processes which occur during relaxation at the low temperature and therefore needs to be extended.

Another advantage of the presented method is the suppression of humidity effects: almost all fit parameters lie within the confidence bounds of each other for both tested RH values. This is a common result for the use of TCO [17]. H_2_ is the only gas where humidity causes significant deviations. We have already started further work to better understand the reaction mechanisms in dry and humid air. The measurement results clearly show that the deliberately simplified model we apply to the sensor does cover most, but not all, observed effects. For CO, H_2_ and NH_3_, the response follows the predicted model with a correlation coefficient close to 1, as well for the $\tilde{k}$ as for the 1/τ parameter. The deviation at low NH_3_ concentration is likely to be caused by errors in gas generation. For benzene, the simplified physical model cannot be applied directly, but requires an empirical extension as described above.

Optimizing the temperature cycle (the last 10 s of all three low-temperature phases are not used in any of the evaluations), one measurement per minute can be conducted. The presented results define a facile technique for the calibration of gas sensor elements for various gases in a high concentration range at minimal cost, that is, with minimal number of calibration points.

## 5. Conclusions

We have shown that the previously presented simple model for MOS sensors based on a single grain–grain boundary offers two new methods for gas quantification with a model-based TCO to obtain linear calibration curves over the full investigated range of concentrations covering 3 to 4 orders of magnitude, which is exceptional for MOS gas sensors. Both methods can be implemented with completely independent algorithms, using a simple rule for switching between both methods. In addition, pattern recognition methods can be applied using the same features to allow identification of the gases. Since the method can be traced back to physical quantities via the model, it is more comprehensible, and therefore credible, compared to highly complex black-box data-based models. Especially for traceable calibration linear, physically motivated models are highly preferable to make use of standard metrology methods, e.g., for determining the limit of detection (LOD) and limit of quantification (LOQ). IUPAC recommendations, for example, have high requirements [36] for being applied to measurement data; future developments here could help to use the full information coming from TCO by also considering more complex data structures. Furthermore, a prerequisite for the calibration of every single gas sensor for a dedicated application, which might be needed for proper results, is the availability of a simple and general correlation between the target concentration and the sensor signal.

We have also shown that our model is able to predict key features of the TCO, but there are still some effects missing, e.g., the appearance of a second relaxation process especially for high concentrations. This might be caused by a changing donor density, but further experiments are required to elucidate the relevant processes and expand the model.

## Figures and Tables

**Table 1:** Overview of the measured gases, their concentrations and the used quantification method.

Gas	Concentrations Measured	$\tilde{k}$-Evaluation	τ-Evaluation
CO	0.01–79.8 ppm	0.01–0.1 ppm	0.1–5 ppm
H_2_	0.01–25 ppm	0.01–0.5 ppm	0.5–25 ppm
NH_3_	0.01–116 ppm	0.01–1 ppm	1–50 ppm
C_6_H_6_	0.01–7.9 ppm	0.01–0.1 ppm	0.1–7.9 ppm

**Table 2:** Fitting parameters for all gases using the $\tilde{k}$-evaluation.

Gas	Humidity	Fit Function	a	b	$R^{2}$
CO	40%RH	f(x)=a⋅x	1939	-	0.9851
-	50%RH	-	1995	-	0.9252
H_2_	40%RH	f(x)=a⋅x	393.1	-	0.9986
-	50%RH	-	351.9	-	0.9983
NH_3_	40%RH	f(x)=a⋅x	108.5	-	0.9381
-	50%RH	-	108.9	-	0.9606
C_6_H_6_	40%RH	$f\left(x\right)=a\cdot x^{b}$	569.9	0.4065	0.9835
-	50%RH	-	691.1	0.4935	0.9938

**Table 3:** Fitting parameters for all gases using the τ-evaluation.

Gas	Humidity	Fit Function	a	b	$R^{2}$
CO	40%RH	f(x)=a⋅x	0.3827	-	0.9874
-	50%RH	-	0.365	-	0.9883
H_2_	40%RH	f(x)=a⋅x	0.1317	-	0.9972
-	50%RH	-	0.1167	-	0.9955
NH_3_	40%RH	f(x)=a⋅x	0.03078	-	0.9888
-	50%RH	-	0.03224	-	0.9960
C_6_H_6_	40%RH	$f\left(x\right)=a\cdot x^{b}$	0.3358	0.3977	0.9963
-	50%RH	-	0.3114	0.3987	0.9942

## Supplementary Materials

The following are available online at http://www.mdpi.com/1424-8220/18/3/744/s1, Figure S1: Heater temperature calculated from the heater resistance around the temperature step from 450 °C to 150 °C.
