# Supplementary material for: Facile Quantification and Identification Techniques for Reducing Gases over a Wide Concentration Range Using a MOS Sensor in Temperature-Cycled Operation
Source: Sensors (Basel). 2018 Mar 1;18(3):744. doi: 10.3390/s18030744 (PMC5876710; doi:10.3390/s18030744)
Supplement: Supplementary file 1 [file sensors-18-00744-s001.pdf]

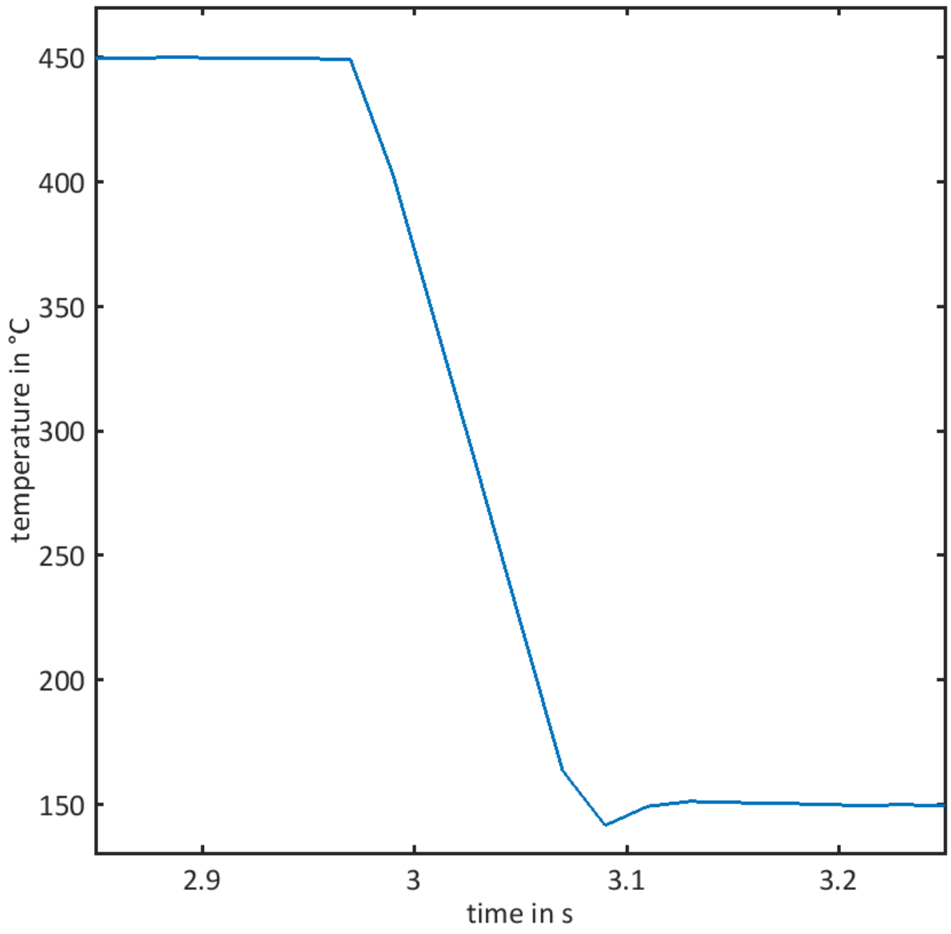

Figure S1: Heater temperature calculated from the heater resistance around the temperature step from 450 °C to 150 °C.
